# COVID-19 in Brazil: spatial risk, social vulnerability, human development, clinical manifestations and predictors of mortality – a retrospective study with data from 59 695 individuals

**DOI:** 10.1017/S0950268821000935

**Published:** 2021-04-23

**Authors:** Jussara Almeida Oliveira Baggio, Michael Ferreira Machado, Rodrigo Feliciano do Carmo, Anderson da Costa Armstrong, Alan Dantas dos Santos, Carlos Dornels Freire de Souza

**Affiliations:** 1 Department of Medicine Federal, University of Alagoas, Arapiraca, AL, Brazil; 2 Post-graduation Program in Family Health, Department of Medicine Federal, University of Alagoas, Arapiraca, AL, Brazil; 3 Postgraduate Program in Health and Biological Sciences, Federal University of Vale do São Francisco (UNIVASF), Petrolina, Pernambuco, Brazil; 4 Postgraduate Program in Biosciences, Federal University of Vale do São Francisco (UNIVASF), Petrolina, Pernambuco, Brazil; 5 Federal University of Sergipe, Sergipe, Brazil

**Keywords:** Coronavirus disease, COVID-19, epidemic, epidemiology

## Abstract

Brazil ranks second in the number of confirmed cases of COVID-19 worldwide. In spite of this, coping measures differ throughout the national territory, as does the disease's impact on the population. This cross-sectional observational study, with 59 695 cases of COVID-19 registered in the state of Alagoas between March and August 2020, analysed clinical-epidemiological variables, incidence rate, mortality rate, case fatality rate (CFR) and the social indicators municipal human development index (MHDI) and social vulnerability index (SVI). Moran statistics and regression models were applied. Logistic regression analysis was applied to determine the predictors of death. The incidence rate was 1788.7/100 000 inhabitants; mortality rate was 48.0/100 000 and CFR was 2.7%. The highest incidence rates were observed in municipalities with better human development (overall MHDI (*I* = 0.1668; *p* = 0.002), education MHDI (*I* = 0.1649; *p* = 0.002) and income MHDI (*I* = 0.1880; *p* = 0.005)) and higher social vulnerability (overall SVI (*I* = 0.0599; *p* = 0.033)). CFR was associated with higher social vulnerability (SVI human capital (*I* = 0.0858; *p* = 0.004) and SVI urban infrastructure (*I* = 0.0985; *p* = 0.040)). Of the analysed cases, 55.4% were female; 2/3 were Black or Brown and the median age was 41 years. Among deaths, most were male (919; 57.4%) and elderly (1171; 73.1%). The predictors of death were male sex, advanced age and the presence of comorbidities. In Alagoas, Brazil, the disease has undergone a process of interiorisation and caused more deaths in poorer municipalities. The presence of comorbidities and advanced age were predictors of death.

## Background

The first case of coronavirus disease 2019 (COVID-19), a respiratory disease caused by the novel severe acute respiratory syndrome coronavirus 2 (SARS-CoV-2), was described in December 2019 in Wuhan, China [1]. It quickly spread throughout the world and reached pandemic status on 11 March 2020 [2].

According to the World Health Organization, by 15 March 2021, 119.9 million cases of the disease had been registered worldwide, with 2.6 million deaths [3]. In Brazil, the first case was confirmed on 26 February 2020, in the city of São Paulo [4]. Since then, the country has accumulated 11.4 million of cases and more than 278 000 deaths, holding second place in the global ranking of affected countries [3].

In addition to this scenario, Brazil is a continent-sized country with great socioeconomic differences, which can lead to different evolution between the states of the federation [5–7]. In Alagoas, the first confirmed case of COVID-19 occurred on 8 March 2020, in a 42-year-old man who had returned from a trip to Italy. The disease initially concentrated in the capital, and from there it subsequently began to spread throughout the state, a dynamic that also occurred in other states of Brazil [8, 9]. Currently, COVID-19 is present in every municipality of Alagoas [10].

Thus, coping with the disease requires an expanded understanding of the dynamics of the spread of COVID-19 in each state, the epidemiological profile and the associated factors, with a focus on planning actions and making decisions. Therefore, the current study aimed to analyse the spatial dynamics of COVID-19 in Alagoas, Brazil, and its relationship with living conditions, to describe the clinical and epidemiological profile and to identify predictors of mortality in the population.

## Methods

### Study design, population and period

A cross-sectional observational study was carried out with 59 695 cases of COVID-19 recorded in the state of Alagoas between March and August 2020. The criterion adopted for confirmation was a positive test (reverse transcription-polymerase chain reaction or serology) for SARS-CoV-2.

### Study location

**Fig. 1.**
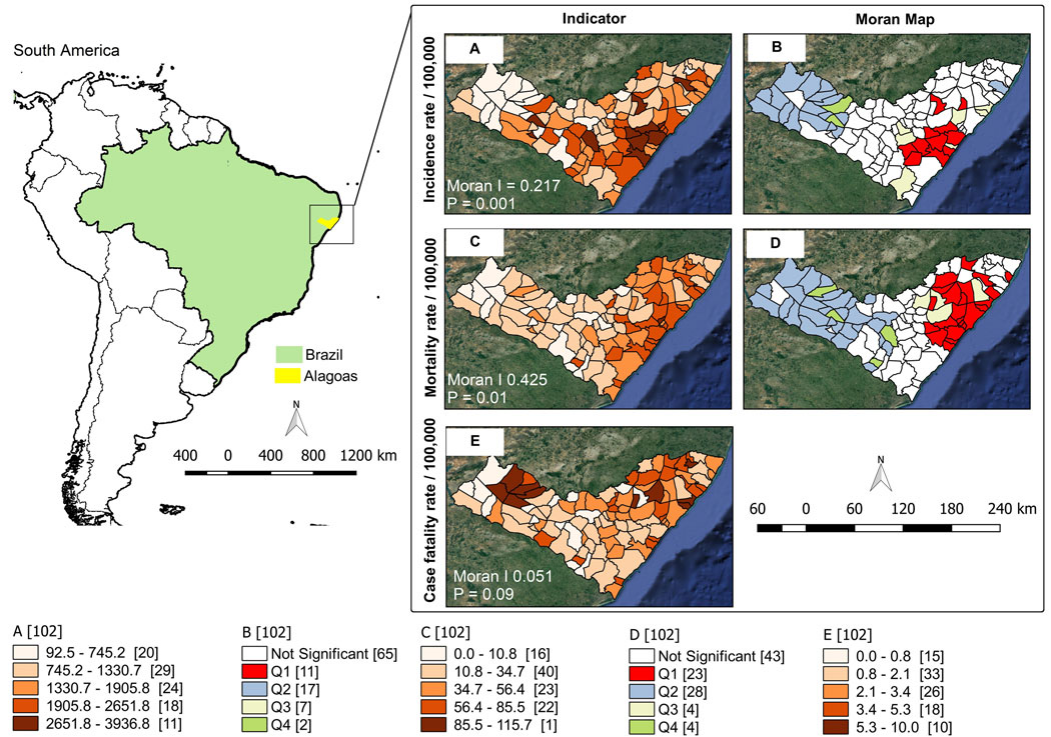
Spatial distribution and risk areas for COVID-19, Alagoas, Brazil, 2020.

The study was carried out in the state of Alagoas, which is located in the Northeast Region of Brazil that has an estimated population of 3.3 million inhabitants, distributed in 102 municipalities (Fig. 1) [11].

### Study variables and data sources

The variables were grouped as follows:

**Group 1**: Comprising the following clinical-epidemiological variables: municipality of residence of the COVID-19 case, age, sex, ethnicity, clinical manifestations, associated comorbidities and clinical outcome.

**Group 2:** Comprising the following epidemiological indicators: cumulative incidence rate of COVID-19 per 100 000 inhabitants, accumulated mortality rate of COVID-19 per 100 000 inhabitants and proportion of fatal cases.

The following equations were used to calculate the indicators:

- COVID-19 incidence rate:
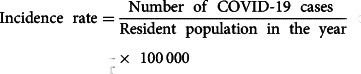
- Mortality rate due to COVID-19:
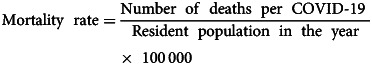
- Case fatality rate (CFR)
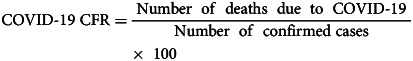

**Group 3:** This group was composed of two social indicators and their respective sub-indices, namely, the municipal human development index (MHDI) and the social vulnerability index (SVI). The MHDI is a composite indicator to assess the degree of human development in a given location. It comprises three dimensions (longevity, education and income), and the index ranges from 0 to 1. The closer to 1, the greater the degree of human development [12]. The SVI, in turn, estimates the degree of vulnerability and social exclusion to which a population is exposed. It is also composed of three dimensions (urban infrastructure, human capital and income and work), and it ranges from 0 to 1; the closer to 1, the greater the degree of social vulnerability [13].

All data related to COVID-19 cases were extracted from the state's public database through http://www.dados.al.gov.br/dataset/painel-covid19-alagoas. Population data were extracted from the Brazilian Institute of Geography and Statistics (IBGE) through https://sidra.ibge.gov.br/home/pms/brasil. Finally, social indicators were obtained from the Human Development Atlas (http://atlasbrasil.org.br/2013/pt/) and the Social Vulnerability Atlas (http://ivs.ipea.gov.br/index.php/pt/sobre), respectively.

### Statistical analysis

After collection, the databank underwent initial evaluation to correct possible inconsistencies. Statistical analysis of the data was subsequently performed with the following two steps:

- Analysis of the spatial dynamics of COVID-19 and high-risk areas

In this step, epidemiological indicators were analysed. For this, global and local Moran statistics were used. The global index provides a general measure of spatial association, whose expression and calculation consider a proximity matrix of order 1. The index varies between −1 and +1, where values equal to zero indicate the absence of spatial autocorrelation, and values close to +1 and −1 indicate the existence of positive or negative spatial autocorrelation, respectively [14].

Once the global dependence was verified, the Local Index of Spatial Association (LISA) was calculated. Based on LISA, the municipalities are positioned in the quadrants of the Moran scattering diagram in the following manner: Q1 (high-high), municipalities where the attribute value and the average value of the neighbours are above the average of the set and which are, therefore, considered highest priority for intervention; Q2 (low-low), the attribute value and the average of the neighbours are below the average of the set; Q3 (high-low), attribute value is greater than that of neighbours and the average of neighbours is less than that of the set and Q4 (low-high), the attribute value is less than that of neighbours and the average of neighbours is greater than the average of the set. The municipalities classified as high-low and low-high have intermediate priority [14]. Finally, choropleth maps were generated to present the results.

For the association between social and epidemiological indicators, Moran's bivariate spatial correlation was used.

This step made use of Terra View software (version 4.2.2, Brazilian Space Research Institute, São José dos Campos, SP, Brazil), GeoDa software version 1.10 (Center for Spatial Data Science, Computation Institute, University of Chicago, Chicago, IL, USA) and QGis (version 2.14.11 Open Source Geospatial Foundation, Beaverton, OR, USA).

- Demographic and epidemiological profile and factors associated with mortality

For this analysis, individuals were divided into two groups, deaths and survivors. Continuous variables were represented using measures of central tendency and dispersion (median and interquartile range) and categorical variables were represented as frequencies (absolute and relative). Fisher's exact test or the chi-square test was used for categorical variables, as appropriate. The Mann–Whitney *U* test was used to compare continuous variables between two categories. In order to identify factors associated with COVID-19 mortality, univariate and multivariable analyses were performed. The variables sex, advanced age (⩾60 years) and comorbidities (cardiovascular diseases, diabetes mellitus, systemic arterial hypertension, obesity, chronic respiratory disease and chronic kidney disease) were tested. In the regression models, the odds ratio (OR) was calculated considering a 95% confidence interval (CI) and a significance level of 5%.

### Ethical aspects

This study used secondary data in the public domain, where it is not possible to identify the subjects. For this reason, Research Ethics Committee approval was waived.

## Results

### COVID-19 spatial dynamics and high-risk areas

On 1 August 2020, the cumulative incidence rate was 1788.7/100 000 inhabitants; the mortality rate was 48.0/100 000 and the CFR was 2.7%. The rates of incidence (*I* = 0.217; *p* = 0.001) and mortality (*I* = 0.425; *p* = 0.001) showed global spatial dependence, with the highest values concentrated in the central (agreste) and eastern (metropolitan) regions of the state. The municipalities of Boca da Mata (3936.8/100 000), Marechal Deodoro (3641.5/100 000) and Olho D’água das Flores (3319.8/100 000) had the highest incidences of the disease. In the Moran scattering diagram, 11 municipalities were classified as high-high, all located between the central and eastern regions of the state. These 11 municipalities were responsible for 9956 cases (16.7% of all records in the state), and they had an average incidence rate of 2735.9/100 000 inhabitants (Fig. 1).

Regarding the mortality rate, the three highest ranking cities were Satuba (115.7/100 000), Coqueiro Seco (85.5/100 000) and Jequié da Praia (77.7/100 000). On the Moran map, 23 municipalities were considered priority. These municipalities were responsible for 1081 deaths (67.5% of the state's records), and they had an average mortality rate of 62.7/100 000 inhabitants. The CFR did not show global spatial dependence (*I* 0.051; *p* = 0.09); consequently, local Moran statistics were not applied. The highest CFRs were registered in Jundiá (10.0%), Paripueira (8.5%), Maravilha (7.7%) and Poço das Trincheiras (7.5%); the first two are located in eastern Alagoas and the latter two are in the sertão region (Fig. 1).

Social indicators associated with the highest epidemiological indicators were also identified. The highest incidence rates were observed both in municipalities with higher human development (overall MHDI (*I* = 0.1668; *p* = 0.002), MHDI education (*I* = 0.1649; *p* = 0.002) and MHDI income (*I* = 0.1880; *p* = 0.005)) and in those with higher social vulnerability (overall SVI (*I* = 0.0599; *p* = 0.033)). Similar results were observed for mortality rate, with one additional indicator (SVI human capital (*I* = 0.0573; *p* = 0.046)). CFR was associated with greater social vulnerability in two dimensions (SVI human capital (*I* = 0.0858; *p* = 0.004) and SVI urban infrastructure (*I* = 0.0985; *p* = 0.040)) (Supplementary material 1).

### Demographic profile and age distribution

**Fig. 2.**
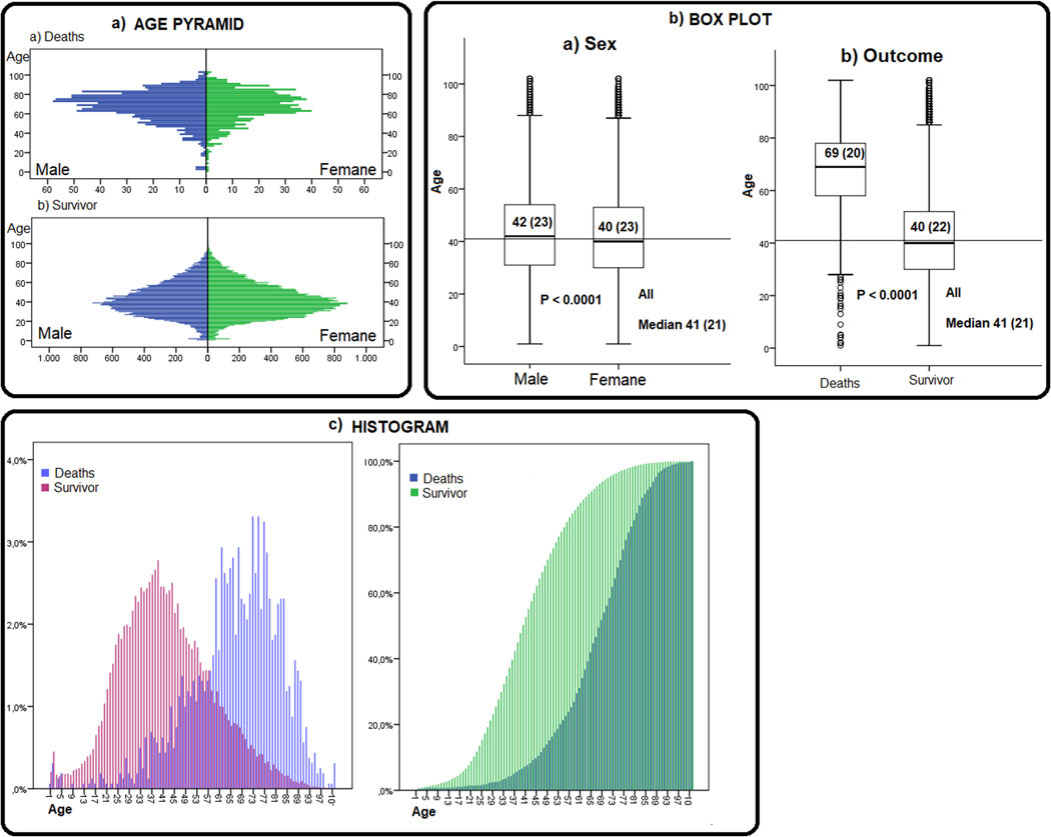
Age distribution of COVID-19 patients, Alagoas, Brazil, 2020.

Of the cases analysed, 55.4% (*n* = 33 087) were female. Considering the deaths, the proportion is inverted; 57.4% of the deaths were male (*p* < 0.0001). Individuals who were Brown and Black accounted for 2/3 of the records (Brown: *n* = 37 855, 63.4%; Black: *n* = 2756, 4.6%). The median age of the population was 41 years (IQR = 21); age was higher in the male population (median: 42 years, IQR = 23) than in the female population (median: 40 years, IQR = 23) (*p* < 0.0001). When comparing deaths and survivors, the median age of deaths was 69 years (IQR = 20), and the median age of survivors was 40 years (IQR 22, *p* < 0.0001) (Fig. 2; Table 1).

**Table 1.** Demographic and clinical characterisation of individuals with COVID-19, Alagoas, Brazil, 2020

Variable	Outcome		Total 59 695 (100.0%)	*p* value
	Deaths 1602 (2.7%)	Survivors 58 093 (97.3%)		
*Sex*				
Male	919 (57.4%)	25 689 (44.2%)	26 608 (44.6%)	<0.0001^a^
Female	683 (42.6%)	32 404 (55.8%)	33 087 (55.4%)	
*Ethnicity*				
Yellow	65 (4.1%)	2019 (3.5%)	2084 (3.5%)	<0.0001^b^
White	161 (10.0%)	9498 (16.3%)	9659 (16.2%)	
Indigenous	1 (0.1%)	200 (0.3%)	201 (0.3%)	
Brown	914 (57.1%)	36 941 (63.6%)	37 855 (63.4%)	
Black	128 (8.0%	2628 (4.5%)	2756 (4.6%)	
Not reported	333 (20.8%)	6807 (11.7%)	7140 (12.0%)	
*Age*				
Median (IQR)	69 (20)	40 (22)	41 (22)	<0.0001^c^
⩽18	17 (1.1%)	3345 (5.8%)	3362 (5.6%)	<0.0001^a^
19–44	131 (8.2%)	31 483 (54.2%)	31 614 (53.0%)	
44–59	283 (17.7%)	14 629 (25.2%)	14 912 (25.0%)	
⩾ 60	1171 (73.1%)	8636 (14.9%)	9807 (16.4%)	
*Initial symptoms reported*				
Fever	757 (47.3%)	30 524 (52.5%)	31 281 (52.4%)	<0.0001^a^
Cough	821 (51.2%)	32 101 (53.3%)	32 922 (55.2%)	0.002^a^
Headache	128 (8.0%)	18 857 (32.5%)	18 985 (31.8%)	<0.0001^a^
Dyspnoea	210 (13.1%)	2655 (4.6%)	2865 (4.8%)	<0.0001^a^
Myalgia	82 (5.1%)	7056 (12.1%)	7138 (12.0%)	<0.0001^a^
Odynophagia	26 (1.6%)	5391 (9.3%)	5417 (9.1%)	<0.0001^a^
Diarrhoea	30 (1.9%)	1058 (1.8%)	1088 (1.8%)	0.861^a^

a Fisher's exact test.

b Chi-squared test.

c Mann–Whitney test.

Among the records, 53.0% (*n* = 31 614) were between the ages of 19 and 44 years. When stratified according to outcome, 73.1% (*n* = 1171) were elderly. Histograms and the age pyramid show a concentration of deaths in the highest age groups and in the male sex. On the other hand, survivors are concentrated in younger age groups and in the female population (Fig. 2; Table 1).

Epidemiological indicators also varied according to age and sex. Although the incidence rate was higher in the female population for all age groups analysed, the mortality rate and the CFR were higher in the male population. In the elderly, the mortality rate in men was 1.75 times higher than that observed in women in the same age group (418.2/100 000 and 238.13/100 000, respectively). Considering both sexes, the mortality rate of the elderly was 315.8/100 000, which is 6.6 times higher than that observed in the general population. These effects of age and sex were also observed in the CFR, which increased with age and was greater in men. In elderly men, the CFR reached 14.4%; among elderly women, on the other hand, the CFR was below 10% (9.8%) (Table 2).

**Table 2.** Incidence rate, mortality rate and CFR of COVID-19, according to sex, Alagoas, Brazil, 2020

*Incidence rate per 100 000 inhabitants*									
Age	Male			Female			Total		
	No. of cases	Population	Rate/100 000	No. of cases	Population	Rate/100 000	No. of cases	Population	Rate/100 000
⩽18	1501	569 142	263.7	1861	548 901	339.04	3362	1 118 043	300.7
19–44	13 681	633 993	2157.9	17 933	696 327	2575.37	31 614	1 330 320	2376.4
45–59	6764	238 203	2839.6	8148	279 995	2910.05	14 912	518 198	2877.7
⩾60	4662	159 988	2914.0	5145	210 808	2440.61	9807	370 796	2644.9
Total	26 608	1 601 326	1661.6	33 087	1 736 031	1905.90	59 695	3 337 357	1788.7

*Mortality rate per 100 000 inhabitants*									
Age	Male			Female			Total		
	No. of deaths	Population	Rate/100 000	No. of deaths	Population	Rate/100 000	No. of deaths	Population	Rate/100 000
⩽18	10	569 142	1.8	7	548 901	1.28	17	1 118 043	1.5
19–44	68	633 993	10.7	63	696 327	9.05	131	1 330 320	9.8
45–59	172	238 203	72.2	111	279 995	39.64	283	518 198	54.6
⩾60	669	159 988	418.2	502	210 808	238.13	1171	370 796	315.8
Total	919	1 601 326	57.4	683	1 736 031	39.34	1602	3 337 357	48.0

*CFR (%)*												
Age	Male				Female				Total			
	No. of deaths	No. of survivors	Total cases	CFR	No. of deaths	No. of survivors	Total cases	CFR	No. of deaths	No. of survivors	Total cases	CFR
⩽18	10	1491	1501	0.7	7	1854	1861	0.4	17	3345	3362	0.5
19–44	68	13 613	13 681	0.5	63	17 870	17 933	0.4	131	31 483	31 614	0.4
45–59	172	6592	6764	2.5	111	8037	8148	1.4	283	14 629	14 912	1.9
⩾60	669	3993	4662	14.4	502	4643	5145	9.8	1171	8636	9807	11.9
Total	919	25 689	26 608	3.5	683	32 404	33 087	2.1	1602	58 093	59 695	2.7

### Clinical manifestations, comorbidities and associated factors

The most common clinical manifestations were fever (52.4%; *n* = 31 281), cough (55.2%; *n* = 32 922) and headache (31.8%; *n* = 18 985), with a higher frequency in survivors. On the other hand, dyspnoea was more frequent in individuals who died (*p* < 0.0001). The frequency of diarrhoea was similar between groups (*p* = 0.861) (Table 1). In univariate analysis, the risk factors for mortality were male sex (OR = 1.69 (95% CI 1.53–1.87)), elderly age (OR = 15.55 (95% CI 13.9–17.41)), cardiovascular disease (OR = 4.28 (95% CI 3.70–4.28)), diabetes mellitus (OR = 8.43 (95% CI 7.49–9.49)), chronic respiratory disease (OR = 3.28 (95% CI 2.45–4.39)), systemic arterial hypertension (OR = 5.62 (95% CI 4.73–6.69)), obesity (OR = 7.73 (95% CI 4.96–12.04)) and chronic kidney disease (OR = 7.52 (95% CI 5.36–10.57)). In multivariate analysis, all variables remained in the model. The factors elderly age and obesity presented the highest ORs (11.87 and 3.22, respectively) (Table 3).

**Table 3.** Logistic regression for identification of death predictors in patients with COVID-19, Alagoas, Brasil, 2020

Risk factor	Outcome		Total (%)	Univariable		Multivariable	
	Deaths *n* (%)	Survivors *n* (%)		OR (CI 95%)	*p* value	OR (CI 95%)	*p* value
*Sex*							
Male	919 (57.4%)	25 689 (44.2%)	26 608 (44.6%)	1.69 (1.53–1.87)	<0.001	1.64 (1.47–1.82)	<0.0001
Female	683 (42.6%)	32 404 (55.8%)	33 087 (55.4%)				
*Age*							
Elderly (⩾60)	1171 (73.1%)	8636 (14.9%)	9807 (16.4%)	15.55 (13.9–17.41)	<0.001	11.87 (10.5–13.36)	<0.0001
Non-elderly (<60)	431 (26.9%)	49 457 (85.1%)	49 888 (83.6%)				
*Cardiovascular disease*							
Reported	234 (14.6%)	2230 (3.8%)	2464 (4.1%)	4.28 (3.70–4.28)	<0.001	1.26 (1.07–1.49)	0.005
Not reported	1368 (85.4%)	55 863 (96.16%)	57 231 (95.9%)				
*Diabetes mellitus*							
Reported	428 (26.7%)	2407 (4.1%)	2835 (4.7%)	8.43 (7.49–9.49)	<0.001	2.82 (2.46–3.24)	<0.0001
Not reported	1174 (73.3%)	55 686 (95.9%)	56 860 (95.3%)				
*Chronic respiratory disease*							
Reported	51 (3.2%)	575 (1.0)	626 (1.0%)	3.28 (2.45–4.39)	<0.001	1.55 (1.13–2.14)	<0.0001
Not reported	1551 (96.8%)	57 518 (99.0%)	59 069 (99.0%)				
*Hypertension*							
Reported	160 (10.0%)	1123 (1.9%)	1283 (2.1%)	5.62 (4.73–6.69)	<0.001	1.75 (1.43–2.14)	<0.0001
Not reported	1442 (90.0%)	56 970 (98.1%)	58 412 (97.9%)				
*Obesity*							
Reported	24 (1.5%)	114 (0.2%)	138 (0.2%)	7.73 (4.96–12.04)	<0.001	3.22 (1.87–5.54)	<0.0001
Not reported	1578 (98.5%)	57 979 (99.8%)	59 557 (99.8%)				
*Chronic kidney disease*							
Reported	41 (2.6%)	202 (0.3%)	243 (0.4%)	7.52 (5.36–10.57)	<0.001	2.55 (1.73–3.75)	0.007
Not reported	1561 (97.4%)	57 891 (99.7%)	59 452 (99.6%)				
*Total*	*1602 (2.7%)*	*58* *093 (97.3%)*	*59* *695 (100.0%)*				

## Discussion

This is the first study to evaluate the spatial dynamics of COVID-19 in the state of Alagoas, Brazil, as well as the relationship with social context, the characteristics of the infected population and the predictors of mortality. In the current study, we observed high incidence and mortality rates in the central (agreste) and eastern (metropolitan) regions of the Alagoas state.

Epidemiological indicators have behaved heterogeneously in the state of Alagoas. In the eastern region of the state, where the capital Maceió and its metropolitan region is located, the disease has a high incidence and mortality; it is expected to be the most populous region and the one where the spread of the disease began. On the other hand, high fatality rates have been observed in the interior.

The relationship between these epidemiological indicators and the context of human development and social vulnerability have shown that incidence and mortality rates, while they are associated with human development, were also associated with social vulnerability. This scenario is related to the expansion process of COVID-19, which first arrived in large urban centres and more developed cities and subsequently spread to smaller and less developed municipalities [7].

A similar study conducted in Ceará also indicates an association between higher MHDI and disease incidence [9]. Furthermore, in Brazil, access to diagnosis of the disease is also associated with better indicators, such as per capita income [15]. The lower incidence in those most vulnerable municipalities may express underreporting of the disease due to the low testing of the population. Thus, assessing the real incidence of COVID-19 in the poorest regions of the country is an even greater challenge, as people lack access to diagnosis and, consequently, appropriate treatment [16–18].

The SVI urban infrastructure takes into account access to basic sanitation and urban mobility services. Thus, the population exposed to unfavourable sanitary conditions has greater difficulty in adopting measures to control the disease, such as social distance and hygiene measures. This context may become even more relevant if we consider the potential transmission of the SARS-CoV-2 virus via the faecal route, although there is still no consensus on the real impact of this type of transmission [19, 20]. These living conditions can increase the levels of emotional stress and the financial impact of the pandemic on the lives of families, making it an even greater challenge to cope with the disease [21].

The relationship between poverty and the burden of COVID-19 has been observed in recent studies. In the USA, greater social vulnerability was associated with a higher lethality rate, which was mainly influenced by the population's socioeconomic status, higher proportion of the population, precarious housing conditions and low access to transportation [22]. Moreover, the COVID-19 pandemic could cause an increase in extreme poverty worldwide. Vos *et al*. projected that a 1% reduction in the growth of the global economy will cause a 1.6–3% increase in the rate of extreme poverty [23]. In Brazil, a study showed that COVID-19 can increase poverty from 17% to 23%, and inequality could increase from 0.55 to 0.59; with the introduction of the Federal Government's Emergency Aid Plan covering informal workers, however, poverty would be reduced to 9.4% [24].

The higher fatality rate in cities with greater social vulnerability can also be related to the local health network. In January 2020, Alagoas had a total of 5881 hospital beds, with an important concentration in the capital (48.73%) [25]. With the exception of Paripueira, which is part of the state's first health region, with patients generally referred to Maceió, the other cities are located in regions with low health infrastructure. The 9th health region, where the cities of Maravilha and Poço das Trincheiras belong, has five ICU beds and 20 exclusive clinical beds for the treatment of COVID-19, and the 3rd health region, where the city of Jundiá belongs, does not have exclusive ICU beds, and it has only 20 clinical beds [26]. These data show that the association between low health infrastructure and low human development index generates a greater number of deaths due to COVID-19.

Regarding the demographic profile of the infected population in Alagoas, it was predominantly female in all age groups; most were Brown or Black and between 19 and 44 years old. These findings were also observed in other Brazilian states [27, 28]. Regarding deaths, the prevalence was higher in males and individuals above 60 years of age. In China, the male sex was the most infected [29, 30]. In Brazil, a study using data from the SIVEP-Gripe system also showed a higher prevalence of males in all reported cases, as well as in cases that died [17]. The relationship between sex and the clinical outcome of COVID-19 may be related to the greater number of comorbidities present in men or to a different immune system response [31]. Further studies are needed to prove this relationship.

Among survivors, the most frequent symptoms were fever, cough and headache, while respiratory symptoms were more frequent in individuals who died. Other studies have found a significant association between shortness of breath and death risk [27, 32] (Macedo, 2020; Soares, 2020). In the respiratory system, angiotensin-converting enzyme 2 expression facilitates the entry of SARS-CoV-2, which mainly infects ciliated cells and type II alveolar cells [33]. After entry, the SARS-CoV-2 virus causes deregulation of the renin–angiotensin–aldosterone system, displacing the axis in favour of angiotensin II, which can generate lung damage due to activation of the inflammatory cascade [34], increased type I collagen with consequent decrease in lung compliance [35] and alveolar cell apoptosis [36]. In spite of this, approximately 5% of cases evolve to critical conditions [37], and the presence of comorbidities seems to be decisive.

In the current study, we found the main predictors of mortality to be male sex, advanced age, cardiovascular disease, diabetes mellitus, chronic respiratory disease, systemic arterial hypertension, obesity and chronic kidney disease. These data corroborate previous studies that indicate the presence of previous comorbidities as an important factor for worsening of the condition [27, 28, 30, 32, 38, 39]. In a study conducted in Pernambuco, Brazil, the presence of cardiovascular diseases, for example, accelerated mortality from COVID-19 by 4 days when compared to individuals without comorbidities [40].

In Brazil, the prevalence of systemic arterial hypertension is 21.4% in the general population, and that of diabetes mellitus is 6.2%. In individuals over the age of 60 years, these prevalence increase to 40% and 19%, respectively [41]. In the Northeast of Brazil, the number of deaths caused by chronic non-communicable diseases has historically been greater than in other regions of the country, which demonstrates the precariousness of controlling these diseases, which would cause an even greater impact of COVID-19 in this region [42].

This study has the following limitations: (i) the database used is in the public domain and it was constructed using COVID-19 notification forms, without adequate standardisation of the variables; (ii) throughout the pandemic, different notification forms have implemented, excluding and/or adding variables and (iii) as it is a new disease, without a clear list of signs/symptoms, it is likely that the less common ones were not identified by patients and registered, especially at the beginning of the pandemic.

In summary, this study shows a high incidence of COVID-19 in municipalities with greater social development, as well as in municipalities with high social vulnerability. The results demonstrate that the disease has undergone a process of internalisation in the state of Alagoas and that it leads to more fatal cases in poorer municipalities. The main predictors of mortality were elderly age, male sex and the presence of comorbidities.

## Data Availability

No additional data were used in the study.

## Supplementary material

For supplementary material accompanying this paper visit http://dx.doi.org/10.1017/S0950268821000935. click here to view supplementary material
