# Supplementary material for: COVID-19 in Brazil: spatial risk, social vulnerability, human development, clinical manifestations and predictors of mortality – a retrospective study with data from 59 695 individuals
Source: Epidemiol Infect. 2021 Apr 23;149:e100. doi: 10.1017/S0950268821000935 (PMC8111197; doi:10.1017/S0950268821000935)
Supplement: Supplementary file 1 [file S0950268821000935sup001.docx]

**Supplementary material 1.** Moran bivariate spatial correlation between epidemiological indicators and indicators of human development and social vulnerability. Alagoas, Brazil, 2020.

| Social Indicator | Incidence rate  I Moran (p value) | Mortality rate  I Moran (p value) | CFR  I Moran (p value) |
| --- | --- | --- | --- |
| MHDI Overall | 0.1668 (p= 0.002) | 0.2182 (p=0.002) | 0.0335 (p=0.152) |
| MHDI Longevity | 0.0243 (p=0.244) | 0.0407 (p=0.086) | 0.0126 (p=0.398) |
| MHDI Education | 0.1649 (p=0.002) | 0.2030 (p=0.002) | 0.0214 (p=0.256) |
| MHDI Income | 0.1880 (p=0.005) | 0.2627 (p=0.002) | 0.0555 (p=0.051) |
| SVI Overall | 0.0599(p= 0.033) | 0.0682 (p=0.036) | 0.0121 (p=0.333) |
| SVI Human Capital | −0.0294 (p= 0.162) | 0.0573 (p=0.046) | 0.0858 (p=0.004) |
| SVI Income and Labor | −0.019 (p=0.302) | −0.0030 (p=0.092) | −0.004 (p=0.074) |
| SVI Urban Infrastructure | −0.0061 (p=0.104) | −0.0242 (p=0.208) | 0.0985 (p=0.040) |

CFR: case fatality rate; MHDI: Municipal Human Development Index; SVI: Social Vulnerability Index.
